# Notes on Genito-Urinary Therapeutics

**Published:** 1883-04

**Authors:** 


### NOTES ON GENITO-URINARY THERAPEUTICS.

(*Continued from page 52.*)

APIS MEL.—Renal pains, soreness on pressure or when stooping.
Frequent sudden attacks of pain along the ureters.

VESICAL.—Great irritation at neck of the bladder, with frequent and burning micturition.
Repeated micturition every few minutes all day.
Difficult urination in children.
Incontinence, with great irritation of the parts.

URINARY PAINS.—Burning and soreness when urinating.
Scalded sensation when urinating.
Frequent micturition, with burning.
Burning and stinging in the urethra.

CHARACTER OF URINE.—Scanty, frequent, and high colored.
Red, bloody, hot, and scanty.
Scanty and fetid.
Reddish brown and scanty; after standing becomes turbid.
Scanty, milky, albuminous.
Dark, with sediment like coffee grounds.
Contains uriniferous tubercles and epithelium.

SEXUAL.—Desire increased.
Frequent and long-lasting erections.
Swelling of testicles, more the right; violent itching and redness of scrotum; sore to touch.
Dropsy of scrotum.

CLINICAL.—Strangury, stricture; retained urine, or inflamed bladder after abuse of cantharides.

ARGENT. N. RENAL.—Acute pain in and about kidneys, extending down ureters to bladder; worse from slightest touch or motion, even a deep inspiration.

VESICAL—Frequent and copious emission of pale urine.
Urging to urinate; urine passes less freely and easily.
Urine passed unconsciously and uninterruptedly.
Scanty and rare emission of dark yellow urine.
Incontinence day and night.

URINARY PAINS.—Urethra is painful, as if closed up.
Sore pain in urethra, even after micturition.

Urine burns while passing, urethra feels as if swollen, with a sensation as if the last drops remained behind. (Alum., Clem., Hepar, Kali b., Thuja.)

(Sensation as if some urine remained in urethra: Agar., Aspar., Ced., Ery. a.)

Cutting pain from urethra to anus.

Ulcerative pain in middle of urethra, as from a splinter.

Inflammation and violent shooting and burning pains in urethra, with increased gonorrhœa.

Stricture; stream of urine divided.

CHARACTER OF URINE.—Urine is dark red; deposits red crystals of urine acid. No albumen.

SEXUAL.—Impotence; erections so weak as to fail when he attempts coition. (*Agn.*, Bar., Calad., Calc., Ign., Nux m., Sep., Sulph., etc.)

Want of desire; organs shriveled. (Penis, Aloe, *Cann. i.*, Ign., Lyc., Merc.; scrotum, Berb., *Crot. t.*, Rhod., Therid., Znc.)

Coition painful; urethra feels as if put on the stretch, or sensitive at its orifice.

Contusive pain (Calc., Dig., Natr. c., Rhod.), with enlargement and hardening of (right) testicles (maybe from suppressed gonorrhœa).

(Testicles hard: *Acon.*, *Agn.*, Alum., *Arn.*, Clem., Lach., Merc., Nux v., Ph. ac., RHOD., Sab., Sil. (r.), SPONG., Strych. (l.).

Priapism, bleeding of the urethra.

Discharge of excoriating pus.

Ulcers on prepuce, covered with pus; later spreading bowl-shaped, with a tallow coating.

(ULCERS on penis: Ars. hyd., Kali iod., MERC., Natr c., *Nitr. ac.*, Psor.

— on glans: Ars., Corall., Nitr. ac., Sep., *Sulph.*, Thuja.

— on prepuce: Arg. n., Ars., Caus., Corall., Hepar, *Merc.*, Nitr. ac., Phos., Sep., *Sulph.*, Thuja.

Ulcers on penis, bleeding: MERC. s.

— — — yellow discharge: *Corall.*

— — — flat: Coral.

— — — itching: Merc. s.

— — — red: Coral., Thuja.

— — — sore: Merc. s.

— — — chancre-like: Hepar, Lac. can., *Merc.*, *Nitr. ac.*, Thuja.

— — — deep: Nitr. ac., Sulph.).

